# Migratory birds, the H5N1 influenza virus and the scientific method

**DOI:** 10.1186/1743-422X-5-57

**Published:** 2008-05-09

**Authors:** Thomas P Weber, Nikolaos I Stilianakis

**Affiliations:** 1 Joint Research Centre, European Commission, T.P. 267, Via Enrico Fermi 2749, I-21027 Ispra, Italy; 2 University Erlangen-Nuremberg, Department of Biometry and Epidemiology, Waldstr. 6, D-91054 Erlangen, Germany

## Abstract

**Background:**

The role of migratory birds and of poultry trade in the dispersal of highly pathogenic H5N1 is still the topic of intense and controversial debate. In a recent contribution to this journal, Flint argues that the strict application of the scientific method can help to resolve this issue.

**Discussion:**

We argue that Flint's identification of the scientific method with null hypothesis testing is misleading and counterproductive. There is far more to science than the testing of hypotheses; not only the justification, bur also the discovery of hypotheses belong to science. We also show why null hypothesis testing is weak and that Bayesian methods are a preferable approach to statistical inference. Furthermore, we criticize the analogy put forward by Flint between involuntary transport of poultry and long-distance migration.

**Summary:**

To expect ultimate answers and unequivocal policy guidance from null hypothesis testing puts unrealistic expectations on a flawed approach to statistical inference and on science in general.

## Background

We welcome the attempt by Flint [1] to base research on the dispersal of H5N1 on a more secure scientific footing, but we think that the approach suggested by him is seriously flawed and counterproductive. According to Flint, the scientific method is identical with null hypothesis testing. However, this approach to statistical inference has many weaknesses and is ill-suited for the task of identifying the mechanisms that are responsible for a natural phenomenon. Instead, we believe that Bayesian approaches are far more productive. Furthermore, in his attempt to show that arguments put forward by us [2] in the debate about the means of dispersal of H5N1 are uninformative, Flint also employs an analogy between poultry transport and long-distance migration that is highly misleading.

## Discussion

### The myth of the scientific method and the trouble with significance testing

Flint urges researchers to apply the scientific method when collecting data and drawing conclusions concerning the dispersal of highly pathogenic H5N1. The term "scientific method", especially used together with the definite article, suggests a unitary and well-defined process. We think that Flint's ideas are far too strict and too normative and probably even harmful. Flint takes a very narrow approach that identifies the scientific method with the formulation and testing of null hypotheses. This stance can be countered on a historical and methodological level: the scientific method is a historical and philosophical myth and null hypothesis testing is a very feeble and inadequate method.

Flint appears to believe that only the testing of hypotheses, not their discovery, constitutes science and is covered by the scientific method. This is an outdated idea. The philosopher Hans Reichenbach [3] introduced the notions of "context of discovery" and "context of justification" into the study of science. The former belonged to the domain of historians, the latter to philosophers. Unfortunately, this division kept philosophy of science away from nearly everything that is fascinating about science: the challenge and excitement of working towards discovering or producing new phenomena in nature or in the laboratory. Philosophy of science has fortunately moved away from trying to impose narrow norms and methods on scientific practice and today emphasizes the diversity of methods and of the means of discovery and justification [4,5]. The scientific method is what scientists do, not what philosophers or scientists, who think that they are philosophers, think they should do. Science is done by error-prone humans working in complex institutions situated in a messy world and therefore does not produce logically indisputable proofs about natural phenomena – this happens only in trivial cases. It usually offers a robust consensus based on a process of inquiry that builds on and allows for constant scrutiny, re-examination, and revision [6]. This is how science works. People ignore the fact that all results in science, whether experimental or theoretical, are provisional.

If we remain on the level of statistical inference, some of these developments are reflected in the increasing use of Bayesian methods in the life sciences. Null hypothesis significance testing still is the dominant statistical approach in biology. However, this approach has come under attack, because it has many, increasingly acknowledged, problems [7-9]. Significance tests provide information on the probability of finding a specific or more extreme event when the null hypothesis is true; they say nothing at all about the probability of a null hypothesis being true. Null-hypothesis-testing encourages rejection or acceptance of hypotheses, rather than an assessment of degrees of likelihood. The *p *value in null hypothesis testing represents the probability of the data if the null hypothesis is true. We believe that it is far more productive and interesting to ask what the probability of a hypothesis is, given the data. The latter requires a Bayesian approach. It also encourages researchers to investigate more than two polarized hypotheses. In such a context, our study [2] can be seen as attempting to provide a basis for estimating prior probabilities: How likely is the hypothesis that long-distance migrants disperse H5N1 given existing data on immunocompetence of migrants? It is not yet possible to provide a quantitative and definitive answer to this question, but this state of affairs should serve as a motivation for more work.

To base research effort, allocation and policy decisions on null-hypothesis testing and type I errors is unproductive. Null hypotheses fail to capture the complexity of nature by giving undue importance to just one of the hypothesis with which the data may be consistent. There still may be a place for significance testing, but it needs to be supplemented among other things by interpretative caution, confidence intervals, effect sizes and power estimates.

### The role of migratory birds and domestic poultry in the dispersal of H5N1

It is beyond reasonable doubt that transport conditions of poultry cause stress and can have a negative impact on immunocompetence [10]. It is, however, very peculiar and odd to compare involuntary transport of poultry with long-distance migration of wild birds. We find it very hard to make sense of this part of Flint's argument and our reply is thus possibly off the mark. Perhaps Flint wants to say that long-distance migration is such a central part of the life-history of many bird species that they follow some sort of "migratory program" and once they started to migrate, they will not stop. The term "program" is in fact often used in studies concerned with the hormonal and neural control of migratory behavior, but migrants are not automata that carry out their journeys irrespective of environmental or physiological conditions. Modeling has identified the selective forces (for example, strong seasonality) that favor long-distance migration without having to take recourse to obligatory internal, periodic processes [11]. Such physiological processes do play an important role in migration [12], but the existence of such mechanisms does not imply necessity; these mechanisms have to be seen as an outcome of selection for migration, not as a cause of migration. It seems more reasonable to assume that migrants base their decisions – when to depart for a flight, the length of the flight episode, how much energy to store etc. – on their own state (energy reserves, health), the state of the environment (food supply, weather conditions etc.) and the time of year. Health, or more generally condition, is a state variable of central importance for our argument.

Here is not the place to repeat the arguments put forward in [2], but two findings need to be emphasized once more. The results reported by van Gils et al. [13] and Hasselquist et al. [14] suggest that some infected birds may migrate with a reduced speed or refuse to embark on long flights. There are many open questions; especially the findings reported in [14] imply that the interaction of infection and heavy physical work in migratory birds may not lead to a clear cut pattern: birds in good general physical condition may be able to deal both with an infection and long flights, whereas birds in bad condition may not. We nowhere claim that it is completely out of the question that wild migratory birds contribute to the spread of H5N1. The interesting question is to what extent and over which distances wild birds can contribute to this spread.

## Conclusion

Thinking only in terms of accepting or rejecting polarized null hypotheses obstructs debate and research about key questions concerning the spread of H5N1. This method only delivers the appearance of certainty. Following Flint's proposals also entails more serious ethical dilemmas than he suggests. Incorrectly concluding that migratory birds can disperse the virus is not just inefficient. Such a conclusion could be used to support measures that impose high costs on migratory birds – a group of organisms that in any case already suffers severely from environmental degradation and climate change. Policy decisions that affect human health, important economic activities and the existence of threatened species deserve a better foundation than the "scientific" method identified by Flint.

## Competing interests

The authors declare that they have no competing interests.
